# Reef Sound as an Orientation Cue for Shoreward Migration by Pueruli of the Rock Lobster, *Jasus edwardsii*

**DOI:** 10.1371/journal.pone.0157862

**Published:** 2016-06-16

**Authors:** Ivan A. Hinojosa, Bridget S. Green, Caleb Gardner, Jan Hesse, Jenni A. Stanley, Andrew G. Jeffs

**Affiliations:** 1 Institute for Marine and Antarctic Studies, University of Tasmania, Nubeena Crescent, Taroona, Tasmania, 7053, Australia; 2 Universidad Católica del Norte, Facultad de Ciencias del Mar, Departamento de Biología Marina, Coquimbo, Chile; 3 Millennium Nucleus for Ecology and Sustainable Management of Oceanic Islands, Coquimbo, Chile; 4 Leigh Marine Laboratory, Institute of Marine Science, University of Auckland, P.O. Box 349, Warkworth, New Zealand; The Evergreen State College, UNITED STATES

## Abstract

The post-larval or puerulus stage of spiny, or rock, lobsters (Palinuridae) swim many kilometres from open oceans into coastal waters where they subsequently settle. The orientation cues used by the puerulus for this migration are unclear, but are presumed to be critical to finding a place to settle. Understanding this process may help explain the biological processes of dispersal and settlement, and be useful for developing realistic dispersal models. In this study, we examined the use of reef sound as an orientation cue by the puerulus stage of the southern rock lobster, *Jasus edwardsii*. Experiments were conducted using *in situ* binary choice chambers together with replayed recording of underwater reef sound. The experiment was conducted in a sandy lagoon under varying wind conditions. A significant proportion of puerulus (69%) swam towards the reef sound in calm wind conditions. However, in windy conditions (>25 m s^-1^) the orientation behaviour appeared to be less consistent with the inclusion of these results, reducing the overall proportion of pueruli that swam towards the reef sound (59.3%). These results resolve previous speculation that underwater reef sound is used as an orientation cue in the shoreward migration of the puerulus of spiny lobsters, and suggest that sea surface winds may moderate the ability of migrating pueruli to use this cue to locate coastal reef habitat to settle. Underwater sound may increase the chance of successful settlement and survival of this valuable species.

## Introduction

The ability of post-larvae to locate suitable habitat in which to settle is critical to the successful recruitment of many marine species that have a planktonic larval dispersal stage [1]. Settlement and recruitment of larvae play a major role in structuring marine populations and are vital to population persistence [2, 3]. Recent studies have demonstrated that the larvae of many marine species do not disperse by passive drifting as was previously thought, but rather they can actively control their dispersal [4, 5]. Consequently, understanding the sensory abilities and cues used by larvae for actively altering their distribution are critical to the development of realistic dispersal models that have useful applications for the management of economically important species [1, 3, 6].

The southern rock lobster, *Jasus edwardsii*, is distributed across southern Australia and around the coast of New Zealand, and supports a valuable fishery with approximately 6,500 tonnes harvested per year [7]. An individual female can produce between 44,000 and 660,000 eggs each year [8] that hatch into larvae known as phyllosoma. The subsequent distribution of these larvae are influenced by ocean currents and eddies where diurnal vertical migration frequently results in their retention 100’s kilometres offshore from benthic populations [9–11]. After 15–24 months and passing through 11 phyllosoma stages, the larvae metamorphose to post-larvae or pueruli up to 220 km offshore and actively swim shoreward during nights in search of coastal rocky reef habitats in which to settle [12, 13]. The mechanisms and orientation cues that the pueruli use to direct their migration towards the coast are uncertain, but onshore advection in combination with active swimming and guidance by a variety of potential environmental cues have been suggested [14–17]. However, in situ experiments examining active orientation responses of *J*. *edwardsii* pueruli to particular cues have not been reported. The pelagic pueruli (stage 1; sensu schema of Booth [18]) upon reaching the coast progresses its development through two subsequent developmental stages over the following 1–3 weeks until moulting to become a reptant juvenile [9, 19]. During this time pueruli can remain nocturnally active, swimming at night whilst in search of suitable deep crevice shelters to complete their development to juveniles but remaining hidden in nooks, crannies and crevices during daylight hours [18, 20].

Recently, the sensory abilities and the behaviour of settlement stages of a variety of marine organisms have received considerable attention, with growing evidence that underwater sound plays an important role in the onshore orientation of coral reef fishes and the post-larvae of some crab species [5, 21, 22]. Underwater sounds emanating from inshore reefs may be detectable tens of kilometres offshore [23] and could carry biologically significant information about the qualities of the source habitat for those organisms possessing sufficient sensory capabilities [5, 24–26]. The ambient underwater sound that emanates from coastal reefs in New Zealand and Australia is dominated by noises generated by snapping shrimp, sea urchins, fishes and other reef animals, and normally this sound increases in intensity after sunset coinciding with the period when pueruli are actively swimming in the water column [18, 25, 27]. Pueruli of spiny lobsters have arrays of pinnate sensory setae along the antennae that may provide the capacity for sound detection [14, 28–31]. No behavioural experiments demonstrating active orientation to underwater sound have been reported in *J*. *edwardsii* or any other spiny lobster species. However, underwater sound was implicated as a possible cause for more than 4,000 pueruli caught in the seawater intake of a power station on the west coast of New Zealand [14, 32], and recently underwater sound from reefs was found to advance the physiological development of pueruli to juveniles [33]. The aim of this study was to determine the *in situ* orientation response of swimming puerulus of *J*. *edwardsii* exposed to underwater sound from a natural reef.

## Materials and Methods

**Fig 1:**
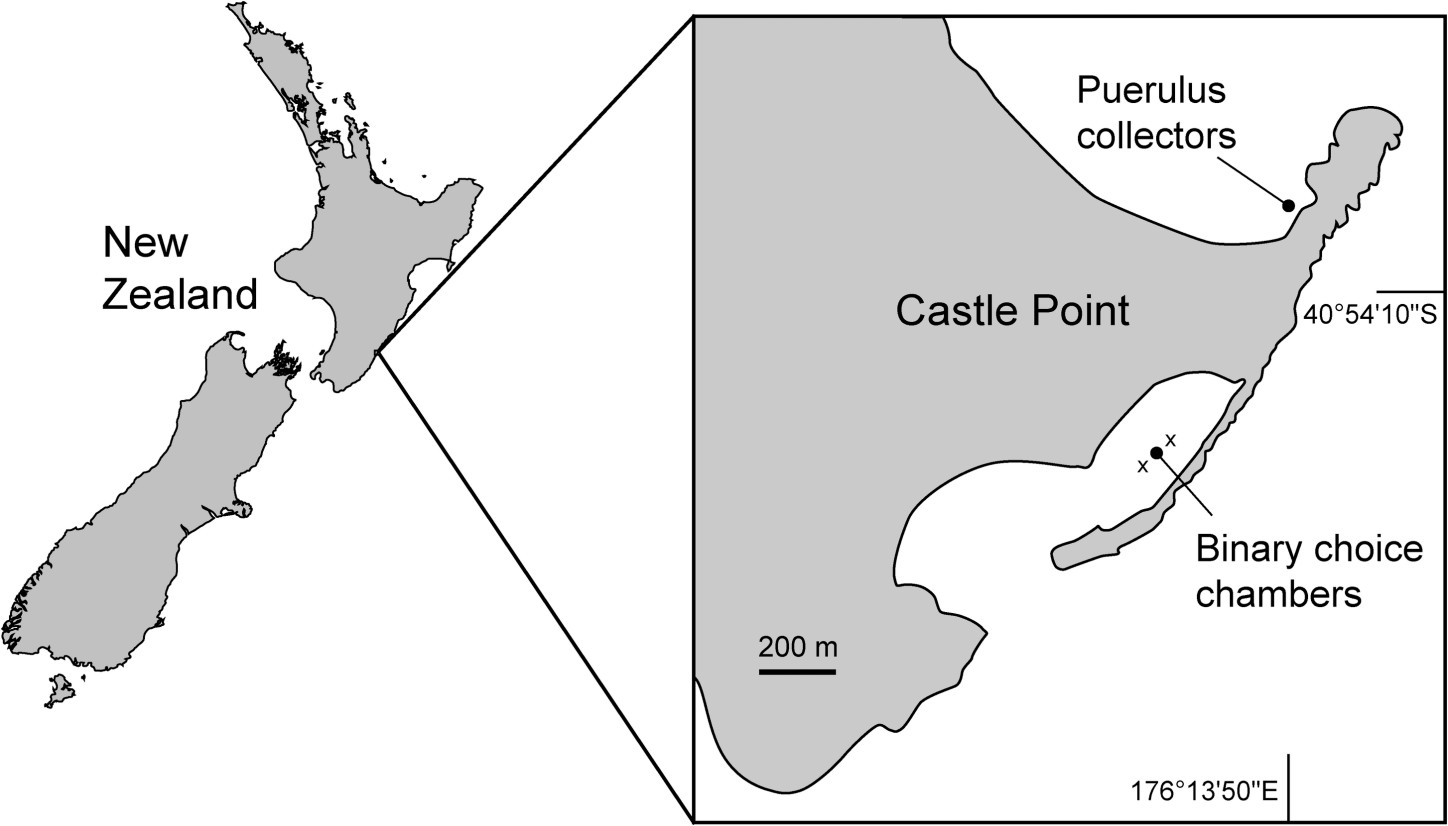
Map of the experimental site. Castle Point experimental sites used for collection of *Jasus edwardsii* pueruli and experimentation with behavioural choice chambers. “X” = positions of underwater speakers. The coastline was extracted from https://www.ngdc.noaa.gov/mgg/shorelines/ and redrawn.

The directional swimming behaviour of pueruli of *J*. *edwardsii* in response to an ambient underwater reef sound was conducted in a field experiment in a sandy lagoon that was protected from waves at Castle Point, New Zealand (40° 54.2' S; 176° 13.8' E) (Fig 1). This experiment was performed on days around the new moon over the austral summers of 2013 and 2015 (7–10 February and 20–23 January, respectively). No specific permissions were required for this location and activity, as it did not involve endangered or protected species. Pueruli were collected using 24 crevice collectors deployed randomly in ~1–2 m water depth during low tide. The crevice collectors are described and illustrated in Booth & Tarring [34] and consist of angled plywood sheets that mimic natural rocky crevice habitat. The collectors were left in running filtered sea water for three weeks before the experiment to leach out any residual chemicals from the plywood used in their construction. Collectors were emptied daily at low tide during daylight hours when pueruli are inactive and hiding in benthic crevices [18]. To empty a collector, a mesh bag was placed around the collector and carried to the shore where pueruli were removed and sorted into different stages following the schema of Booth [18]. Collectors were immediately returned back into position in readiness for collection of nocturnally active pueruli during the following night. Only stage one pueruli were retained and used for experimentation as they were most likely to have arrived in the collectors in the preceding hours of darkness from their pelagic migration. Regardless, stage one pueruli taken from collectors are known to continue actively searching at night for suitable habitat to complete their settlement [18, 20]. Collected stage one pueruli were held individually in 40-ml floating jars with perforated lids within a 60-l drum with aerated seawater until they were used in the experiment later that day. At sunset, the pueruli were transported within a 20-l bucket to the experimental site for trials during the night (Fig 1).

**Fig 2:**
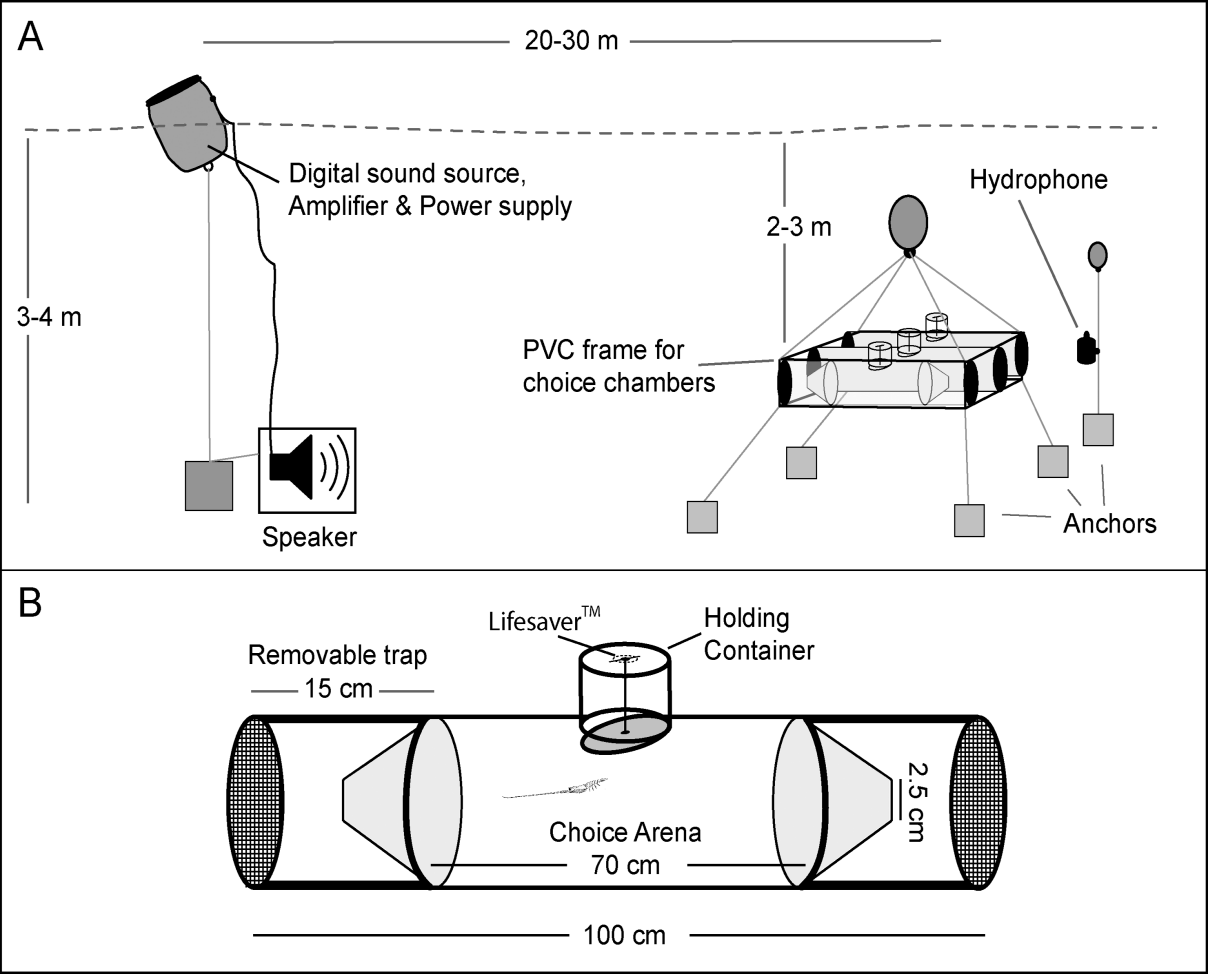
Experimental set up and choice chamber. (A) Schematic of experimental set up, and (B) behavioural choice chamber design.

**Fig 3:**
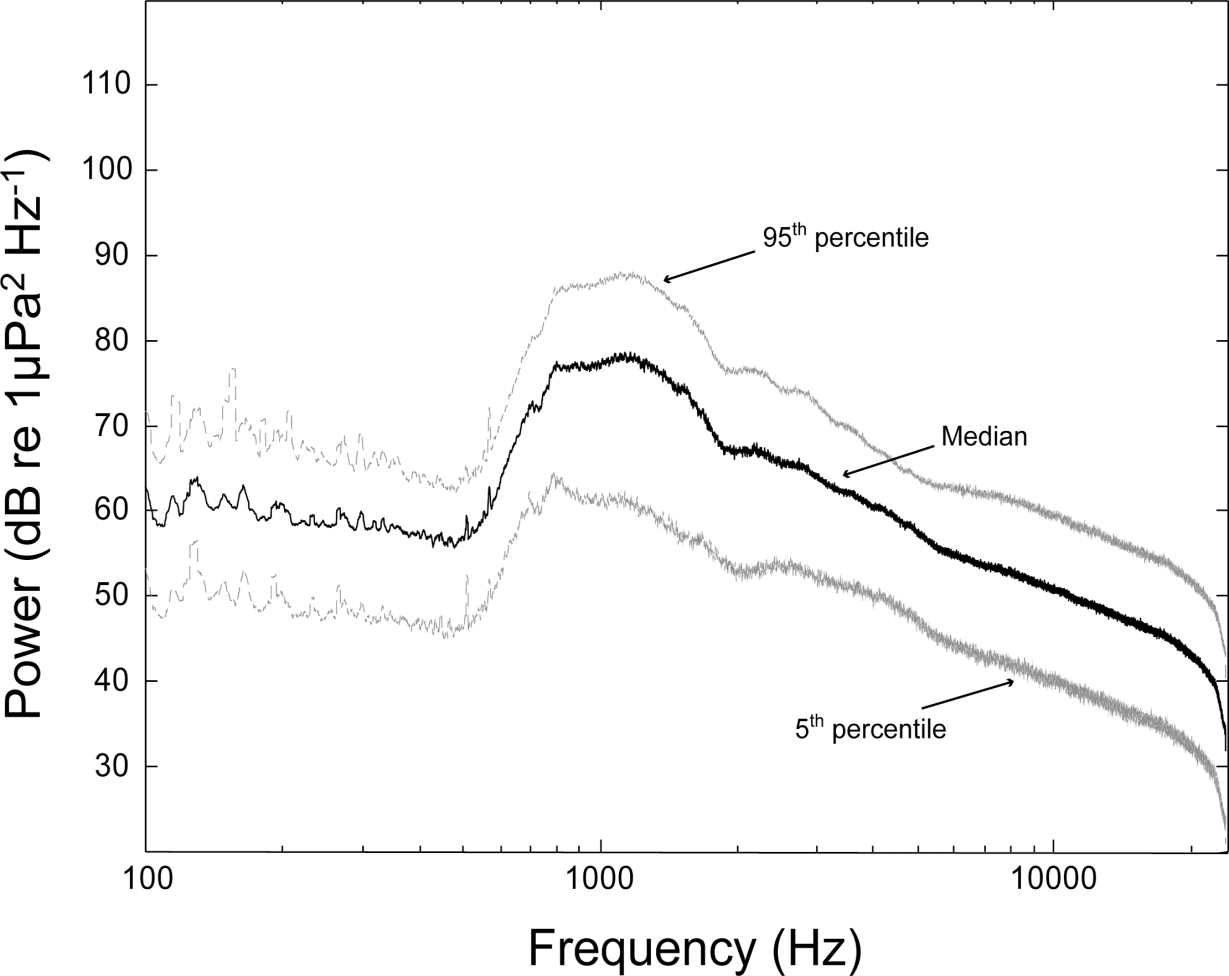
Broadcast sounds from a natural reef habitat. Power spectra of the broadcast sounds from a natural reef habitat during the summer replayed in the *Jasus edwardsii* behaviour field experiment. Solid black line represents the median spectra for the 6 minute recording and dashed grey lines represent the spectral variability (5 & 95 percentiles) determined from a series of non-overlapping 10 s duration windows (S1 Sound File).

At the experimental site, replicate binary choice chambers were deployed in parallel at 2–3 m water depth and 1 m from the sandy seafloor (Fig 2A). The entrance to the lagoon is more than 600 m away so there was no directional tidal flow at the experimental site. Three to six replicate chambers were deployed at the experimental site each night depending on the number of pueruli obtained from the collections each day. The binary choice chambers were similar in design to that described by Radford et al. [22] and consisted of a transparent acrylic plastic tube (9 cm internal diameter, 100 cm long) with detachable trap ends (each 9 cm diameter, 15 cm long) covered with 500 μm plastic mesh (Fig 2B). A square frame made of transparent acrylic plastic sheet held three choice chambers spaced 20 cm apart. The choice chambers were oriented parallel to the rocky reef running along one side of the lagoon to ensure there was no unequal influence of cues from reef habitat at either end of the choice chamber. An underwater audio speaker (Lubell Labs Inc., LL9642; 250 Hz–20 kHz, 170 dB re 1 μPa @ 1 m) was operated with a digital sound source (Sony MP3 player), an amplifier and a power supply housed inside a sealed drum floating above the speaker (Fig 2A). This speaker system was positioned at ~20–30 m from the end of the choice chambers with the position alternated on either end of the choice chamber between nights to remove directional bias (“X” in Fig 1). The ambient underwater reef sounds used in the experiments were recorded in northeastern New Zealand (36° 15' S, 174° 47' E) during the spring at dusk over two nights on a new moon, using a remote underwater recording system that consisted of a calibrated HTI-96-MIN omnidirectional hydrophone (High Tech Inc., flat frequency response over the range of 10–24,000 Hz) connected to a digital recorder (Edirol R09HR 24-bit recorder; sampling rate 48 kHz, Roland Corporation, Japan), contained in an underwater housing. The hydrophone was placed ~1 m off the seafloor in 23–25 m of water, and 30 m away from the margin of the coastal fringing rocky reef. There were no anthropogenic sources of noise such as large vessels or recreational boats visible in the area at the time of recording. All recordings were conducted in near calm conditions (< 0.5 m wave height and < 2.6 m s^-1^ wind speed; Climate Station, Leigh Marine Laboratory) so sound was primarily biological in origin rather than from waves or wind. Three typical 2 min sound sequences from the original habitat recording were randomly selected from a total of two hours of recording taken from a coastal reef in northeastern New Zealand on two separate nights and transferred to a MP3 player and used for playback in the experiments (S1 Sound File). Prior to use in the experiments, spectrograms and waveforms of the selected sound sequences were inspected to confirm the absence of anthropogenic or abnormal noise. These three different sound sequences were randomly chosen to avoid pseudoreplication that would have occurred by using a single recording from the reef [35]. There was a consistent peak in the spectra of all sound sequences located at around 1.2 kHz, which Radford et al. [36] assigned to the feeding sounds produced by the sea urchin, *Evechinus chloroticus*, and also higher frequency pulses from the snaps of snapping shrimp (Fig 3) (S1 Sound File) [37, 38].

Deployment of pueruli into the chamber occurred during the night (21:00 to 01:00 hrs NZ Standard Time), whereby a single puerulus (n = 64) was transferred from the 40 ml floating jar to a sealed plastic holding container (400 ml). This container was then placed at the centre of the choice chamber by a diver on snorkel (Fig 2B). Both procedures were conducted with illumination by red light (Kodak Wratten Gelatin Filter #29; >600 nm) which is outside the visible spectrum of spiny lobsters [39]. Each puerulus was remotely released into the choice arena after approximately 20 min, by the automatic opening of the holding container as a result of a dissolving sugar lolly (Lifesaver^TM^; Fig 2B). The choice chamber was surveyed one hour after attaching the holding container, leaving each puerulus ~40 min to make a directional choice by swimming into one of the traps at either end of the tube (Fig 2B). This time period was selected based on preliminary laboratory experiments that had indicated that the pueruli made a choice at around 30 min after release. The position of pueruli in the choice chamber (reef sound or silent side of the chamber relative to the speaker position) was recorded either *in situ* by a diver on snorkel, or by removing the chamber from the frame and observing in which end of the chamber the puerulus was trapped.

Underwater sound was recorded only during the 2015 experimentation with a calibrated remote hydrophone (SoundTrap 202, working frequency range of 0.020–60 kHz) that was located 1 m perpendicular to the choice chambers. This recorded the potential variation in ambient and replayed sound at the experimental site (S2 and S3 Sound Files). The experiment was conducted under different wind speed and tide conditions (ambient conditions) on individual nights, and differences in choices under these conditions were analysed using a logistic regression model [40]. Corresponding tide phases were extracted from Meteorological Service of New Zealand’s tide tables (http://www.metservice.com/) and the wind data from the National Climate Database (http://cliflo.niwa.co.nz/). The logistic regression models measured the relationship between a dichotomous categorical dependent variable (puerulus choice) and independent variables that could be continuous and/or categorical (ambient conditions) by using probability scores as predicted values of the dependent variable [40]. We included as independent variables: the speaker position (“north” or “south”, relative to the chambers; “X” in Fig 1), the tide phases (as “stable” and “changing”), the wind gust direction, and the wind gust speed. The tide phases were included in the analysis to account for any influence of tide current on the swimming direction of the puerulus. The current was considered to be “stable” when experiments were conducted during low or high tides and “changing” when the tide was shifting between low and high. The model was run using a backwards step-down with a bootstrap of 1,000 iterations to determine which independent variables to include in the final model [40, 41]. These analyses were performed with R using the regression modelling strategies (rms) package [41, 42]. Additionally, we tested whether the pueruli were attracted by the artificial source of reef sound during the windy (>25 m s ^-1^) and non-windy days with two simple goodness-of-fit tests using an equal preference for both sides of the choice chamber as the expected frequency [40]. Data were pooled from all nights and were considered as observed frequency in the test [40].

## Results

**Fig 4:**
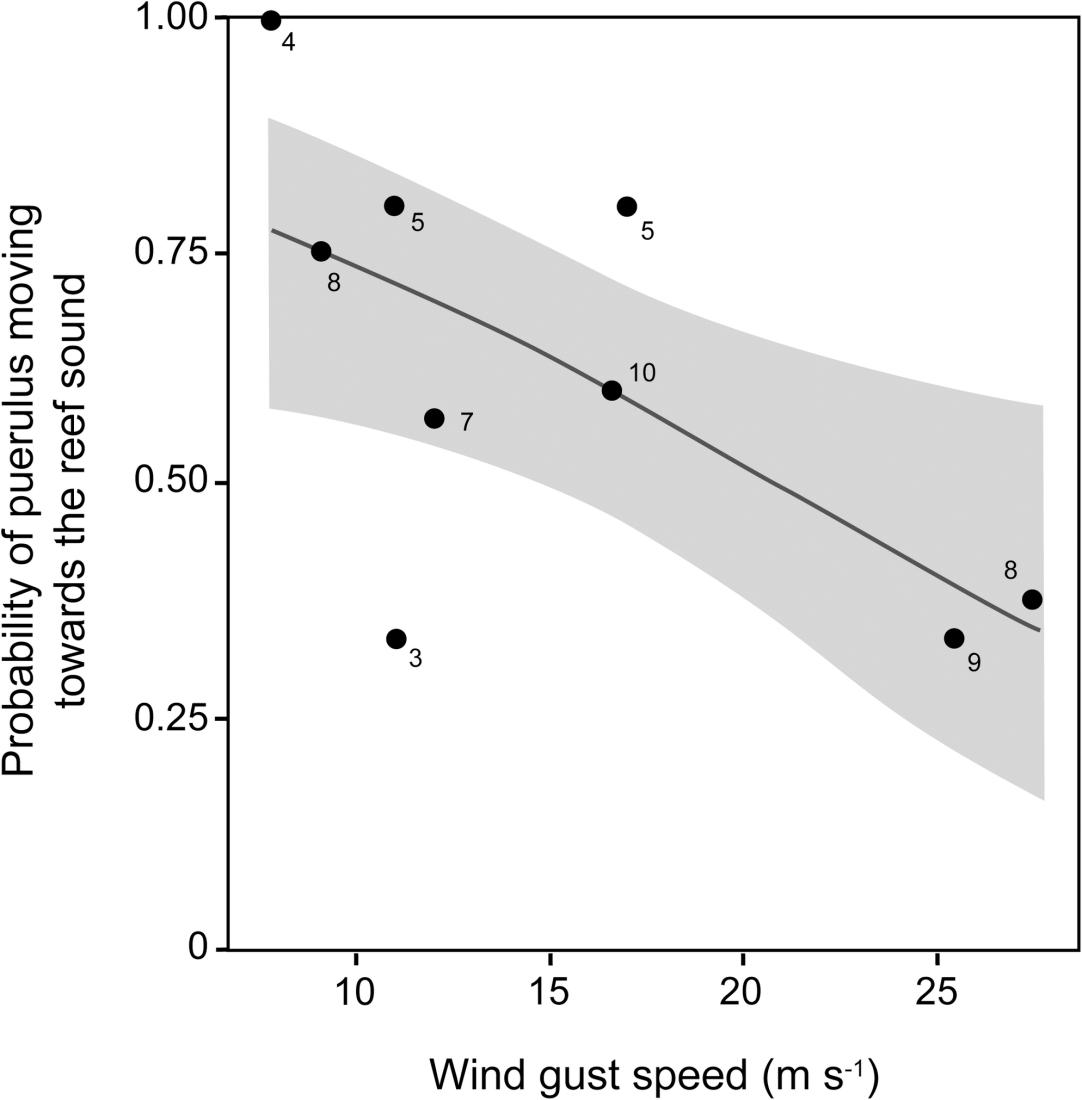
Predicted probability and observed proportion of puerulus choosing to move towards the reef sound for the range of wind gust speeds experienced over the study period. Predicted probability of a *Jasus edwardsii* puerulus choosing to move towards the reef sound moderated by the wind gust speed (m s^-1^), based on a logistic regression (grey area represents the 90% CI) (Wald Z = -2.4; P = 0.02; n = 59). Black dots in the graph represent the observed proportion of the puerulus per night choosing to move towards the reef sound, while the numbers adjacent to the black dots represents the total puerulus sample size used to generate the proportion.

Of the 64 pueruli tested, 59 (92.2%) moved and were trapped into one of the two sides of the experimental chamber. The remaining five hid in the delayed release mechanism so were excluded from analyses. Overall, the majority of the pueruli (n = 35; 59.3%) moved from the choice arena towards the sound, independent of the speaker position (Wald Z = 0.1; P = 0.96; n = 59), wind direction (Wald Z = 0.7; P = 0.47; n = 59) or tide phases (Wald Z = 0.8; P = 0.44; n = 59; Tables 1 & 2), indicating a behavioural choice. However, this selection by puerulus was moderated by the wind gust speed (Wald Z = -2.4; P = 0.02; n = 59; Table 2; Fig 4). Sixtynine percent (n = 29) of the pueruli moved toward the artificial source of reef sound when wind gusts were below 25 m s^-1^ (χ^2^ = 6.1; P = 0.01; n = 42). In contrast, pueruli exhibited no significant preference for either side of the choice chamber (χ^2^ = 1.5; P = 0.23; n = 17) when the wind gust speed was higher than 25 m s^-1^.

**Table 1:** Directional choice of *Jasus edwardsii* pueruli in the behavioural choice chamber (towards or away from the artificial sound), position of the speaker, and ambient environmental variables at the experimental site (timing of low tide, gust wind direction and speed).

Date	Toward Sound (%)	Away from Sound (%)	N	Speaker position	Low tide time	Gust Dir. (Deg)	Gust Speed (m s^-1^)
7/02/2013	75.0	25.0	8	N	21:28	245	8.8
9/02/2013	80.0	20.0	5	S	22:27	314	17
10/02/2013	57.1	42.9	7	N	0:01	30	11.8
12/02/2013	33.3	66.7	9	S	1:52	328	25.7
13/02/2013	37.5	62.5	8	N	3:35	323	27.8
20/01/2015	60.0	40.0	10	N	23:44	208	16.5
21/01/2015	33.3	66.7	3	S	0:26	162	10.8
22/01/2015	80.0	20.0	5	N	1:10	193	10.8
23/01/2015	100.0	0.0	4	S	2:00	40	7.7

**Table 2:** Logistic regression of the directional choice of *Jasus edwardsii* pueruli in relation to the speaker position and environmental variables (tide phase, wind gust direction and wind gust speed).

Model: Pueruli choice = intercept + speaker position + tide phase + wind gust direction + wind gust speed							
	Coef.	S.E.	Wald-Z	Pr(>|Z|)			
Intercept	1.7	0.8	2.2	0.031			
Speaker position	0.0	0.6	0.1	0.956			
Tide phase	0.5	0.6	0.8	0.440			
Wind gust direction	0.0	0.0	0.7	0.465			
Wind gust speed	-0.1	0.1	-2.1	**0.034**			
Model validation: Backwards Step-down							
Deleted	χ^2^	d.f.	P	Residual	d.f.	P	AIC
Speaker position	0.0	1	0.956	0.0	1	0.956	-2.0
Wind gust direction	0.6	1	0.451	0.6	2	0.752	-3.4
Tide phase	0.3	1	0.561	0.9	3	0.823	-5.1
Suggested Model: Pueruli choice = intercept + wind gust speed							
	Coef.	S.E.	Wald Z	Pr(>|Z|)			
Intercept	1.959	0.733	2.670	0.008			
Wind gust speed	-0.094	0.040	-2.370	**0.018**			
Suggested Model Likelihood Ratio Test							
LR (χ^2^)	6.020						
d.f.	1.000						
P	**0.014**						

**Fig 5:**
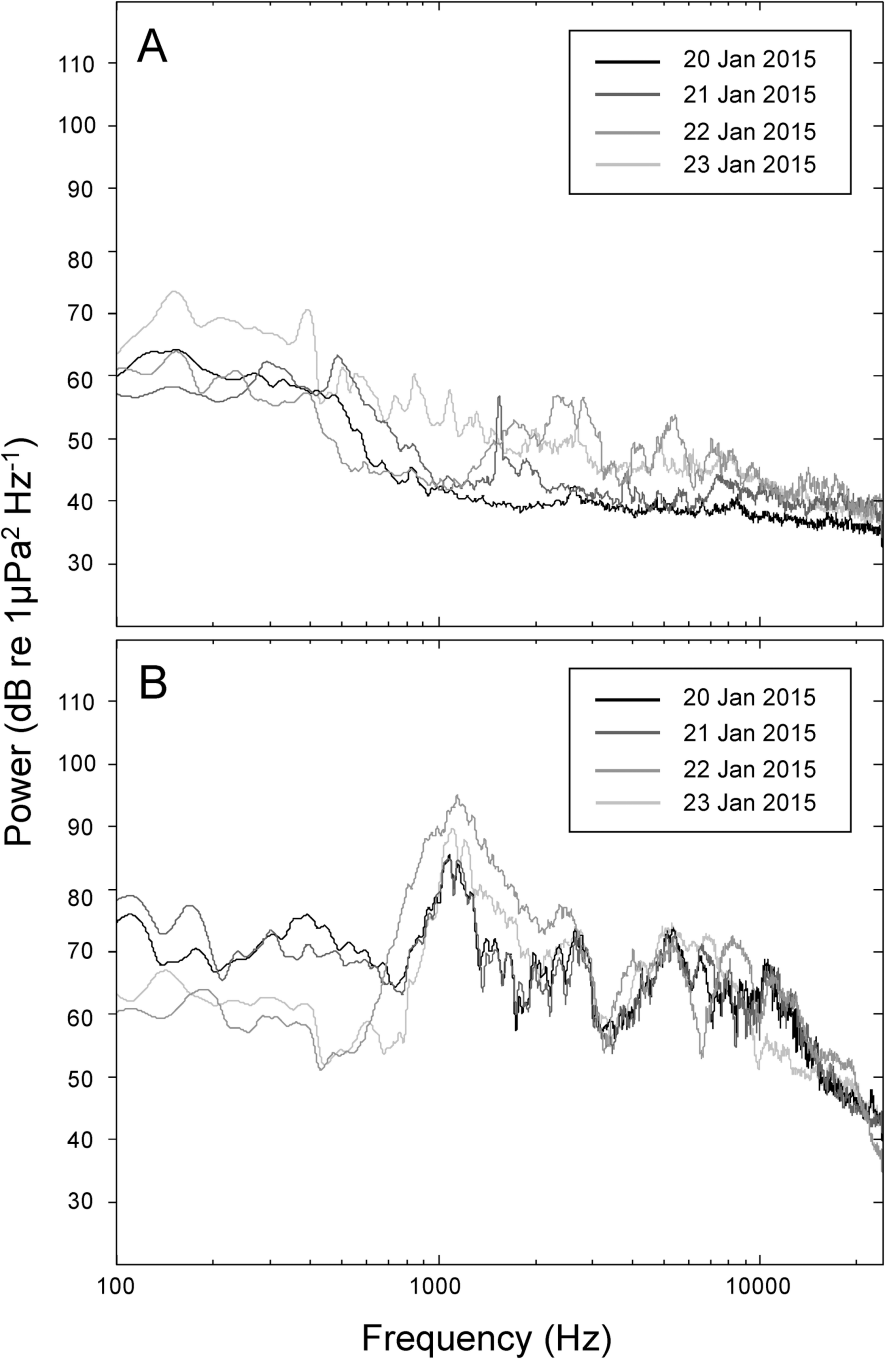
Underwater sounds at the experimental site. Power spectra of underwater sound recorded for each night at the experimental site in January 2015 (A) immediately prior to the artificial source of underwater sound commencing (S2 Sound File), and (B) during the experiments with the artificial source of underwater sound present (S3 Sound File).

The ambient underwater sound recorded at the choice chambers in the absence of replayed reef sound had a similar power spectrum among days, with higher acoustic power at lower frequencies (below 300 Hz) during the summer of 2015 (Fig 5A). In contrast, in the presence of replayed reef sound the sound recorded at the choice chamber had higher acoustic power overall and especially at frequencies around 1.2 kHz that were generally consistent with the overall sound intensity and spectrum of the reef from where the original recordings were taken (Figs 3 and 5B).

## Discussion

A significant majority of pueruli of *J*. *edwardsii* actively moved toward the projected sound of a natural reef, suggesting that they could use reef sound as a cue to find reef habitats in which to settle. Pueruli made directional choices within the confines of a choice chamber within which there would have been a negligible sound pressure gradient, given that there is very low attention of sound propagated in seawater, especially at the lower frequencies that dominated the replayed reef noise [43]. Therefore, it is most likely that the pueruli are sensing and responding to the directionality of the particle velocity component of sound rather than any pressure differential. Similar directional behavioural choices have been reported in the pelagic settlement stages of a wide range of coastal fishes, decapod crabs and coral [21, 22, 31, 44–47]. In our experiment, higher wind velocity reduced the effect of the reef sound on swimming direction of pueruli. This is likely to be due to the increase in abiotic underwater sound at higher wind speed (e.g., >20 m s^-1^) as a result of the disturbance of the sea surface or breaking waves on the nearby reefs [27, 43]. This abiotic sound from wind would have partially overlapped the dominant bandwidth of our projected reef sound, i.e., 50–1100 Hz [27, 43]. Scattered sources of increased sound from the surface of the water would have the potential to mask directional sound emanating from coastal reefs, making it more difficult for pueruli to detect directionality of the replayed reef noise from amongst the background of multidirectional sound [27].

The distribution of the phyllosoma stages of *J*. *edwardsii* is mainly influenced by large scale transport processes, such as currents and eddies, where diurnal vertical migration may result in their retention 100’s of km from benthic populations [10, 11]. The phyllosoma metamorphose to the pueruli as far as 200 km offshore from shallow coastal habitats [48] and the pueruli have been observed rapidly swimming in straight lines at the sea surface at night [49]. There is evidence that the movement of pueruli across the shelf can be influenced by large scale transport processes such as Ekman Current transport associated with along shore winds [50]. However, active swimming by pueruli also appears to play an important role in onshore transport, possibly explaining why in some locations, such as Castle Point, New Zealand, it has not been possible to determine a relationship between puerulus settlement and local environmental variables that would otherwise be associated with passive onshore transport of larvae [51].

Pueruli of *J*. *edwardsii* are a non-feeding (lecithotrophic) stage that depend only on the energy reserves stored during the preceding phyllosoma phase so the duration of the pueruli phase is constrained by these limited energy reserves [52, 53]. Therefore, it seems unlikely that pueruli would rely solely on an orientation cue that is only available during calm conditions, and more likely use a hierarchy of orientation cues, as has been found in settlement stages of other species [5, 54, 55]. In windy conditions, pueruli may be able to use other shoreward cues for orientation such as celestial, hydrodynamic or chemical cues [14–16]. For example, chemical cues derived from suitable settlement habitats were found to elicit a chemotactic response in the pueruli of *Panulirus argus* [15]. Higher concentrations of pueruli of *P*. *cygnus* have been found at the surface of the sea in rough sea conditions, possibly using directional cues from waves, such as Stoke’s drift [56]. Similarly, *in situ* experiments on tethered pueruli of *P*. *argus* found they were orientating in response to wind direction as well as tidal flow [16]. Consequently, it is possible that *J*. *edwardsii* pueruli may use other directional cues at times when strong winds mask underwater sound orientation cues.

The temporal and spatial pattern of settlement of spiny lobster pueruli is difficult to predict, but it is important for estimating future recruitment levels of valuable fisheries for these species [57–59]. Therefore, an ability to determine the effectiveness of physical orientation cues used by pueruli, and to predict the migration pathway of the pueruli from metamorphosis to their eventual settlement locations, has the potential to improve the predictive power of biophysical models [6, 59]. This is likely to be important in spiny lobster because their pueruli have considerable capacity for active migration versus relying on passive transport or weak swimming capabilities alone. For example, the pueruli of *J*. *edwardsii* can swim at velocities around 10–40 cm s^-1^ for sustained periods providing the capacity to move considerable distances shoreward provided effective orientation cues are in use [49, 60].

The experiment in the current study used only underwater sound previously recorded from a reef habitat in which *J*. *edwardsii* lobsters were present in northeastern New Zealand [25, 37] suggesting that pueruli can detect sounds and be attracted by them in certain conditions. However, there is good evidence that differences in underwater sound associated with differences in habitat at their source may be used by pelagic settlement stages of other species to remotely select and orientate their migratory behaviour [44, 61–63]. The research methods used here for pueruli of *J*. *edwardsii* could also be applied to test the effect of different sources of underwater sound from different coastal settlement habitats. Additional research is also required to determine the range offshore over which the underwater sound may provide an effective orientation cue, including confirming the extent to which wind on the sea surface would mask the reliable detection of the sound cue [64]. Previous studies estimating the ranges at which larvae can detect underwater sound have tended to use measures of sensitivity to sound pressure to estimate possible detection ranges, however, this may be inappropriate if, as the results of this study suggest, marine organisms are using particle velocities to detect sound directionality [23]. Improving our understanding of the effective range of underwater sound cues in lobster pueruli will therefore rely on determining both their sensitivity to the particle motion component of the acoustic field and the directional component of particle velocities of underwater reef noise at different distances from source.

In the megalopae of a number of crab species, the habitat type or habitat quality influences settlement behaviour [38, 62]. Habitat type is also known to affect the survival of early benthic stage *J*. *edwardsii* and this could influence spatial patterns in abundance [17]. This is of particular interest with *J*. *edwardsii* because there have been recent dramatic changes in coastal reef communities across part of their range as a result of climate change and the removal of predators of sea urchins [65–68]. Changes in these communities brings marked changes in the soundscape from the reefs [24, 25, 69] and potentially may have some major impacts on the orientation and settlement of the pueruli of *J*. *edwardsii* as occur in other species [44, 61, 62]. For example, the dramatic loss of kelp from reefs and replacement with urchin-dominated barren reef habitat has been associated with very marked differences in the frequency and intensity of the corresponding underwater sounds these different habitats produce [25]. These changes in the important underwater sound cues used by settling pueruli have the potential to greatly influence the successful settlement and recruitment in this spiny lobster species.

Overall, the settlement of *J*. *edwardsii* appears to involve active nocturnal searching [18] where multiple cues from the habitat may be involved to guide the pueruli to reef so that they can locate a hole or crevice in which to settle [17]. Underwater sounds are thought to be able to be detected tens of kilometres offshore [5, 23] so it is feasible that sound could be used by pueruli to orientate their swimming after metamorphosis from the phyllosoma. However, the distance at which this cue may be used by pueruli needs more empirical research. Our experiment demonstrated that pueruli, under certain conditions, can respond to the directionality of underwater sound which strongly suggests that sound can play a role in successful settlement and survival of this valuable species.

## Supporting Information

S1 Sound File Reef sound recorded at North reef and used in the experiments. (WAV)

S2 Sound File Ambient sound recorded at the experimental site before experiments. (WAV)

S3 Sound File Ambient sound recorded at the experimental site during experiments. (WAV)
